# Hyperaldosteronism in Mice Lacking the Distal Polybasic Tract of the γ‐Subunit of the Epithelial Sodium Channel During Sodium Restriction

**DOI:** 10.1111/apha.70228

**Published:** 2026-04-27

**Authors:** Daniel Essigke, M. Zaher Kalo, Matthias Wörn, Xia‐Qing Li, Bernhard N. Bohnert, Anja Schork, Andreas L. Birkenfeld, Thomas Ott, Ferruh Artunc

**Affiliations:** ^1^ Internal Medicine IV, Department of Diabetology, Endocrinology and Nephrology University Hospital Tübingen Tübingen Germany; ^2^ Institute for Diabetes Research and Metabolic Diseases (IDM) of Helmholtz Munich at the University of Tübingen Tübingen Germany; ^3^ German Center for Diabetes Research (DZD) Tübingen Germany; ^4^ Department of Nephrology Peking University Shenzhen Hospital Shenzhen China; ^5^ Max Planck Institute for Biological Cybernetics Tübingen Germany

**Keywords:** edema, epithelial sodium channel, nephrotic syndrome, sodium retention

## Abstract

**Aims:**

The epithelial sodium channel ENaC consists of the subunits α, β, and γ and is activated at an individual channel level by proteolytic processing. Murine γENaC contains a distal polybasic tract 186^RKRK^ mediating proteolytic ENaC activation by serine proteases in vitro. The relevance of ENaC activation at this cleavage site for sodium homeostasis in vivo is unknown.

**Methods:**

Mice were generated carrying a mutation of the distal polybasic tract (RKRK186QQQQ or γENaC^ki/ki^) using CRISP/Cas9. Sodium homeostasis and proteolytic processing of γENaC were investigated under a low sodium diet, pharmacological ENaC blockade, and induction of nephrotic syndrome.

**Results:**

Under control conditions, the response to bolus amiloride was reduced in γENaC^ki/ki^ mice compared to γENaC^wt/wt^ mice. Under a low sodium diet for 4 days, urinary sodium excretion was similarly lowered in both genotypes; however, γENaC^ki/ki^ mice required significantly higher plasma aldosterone concentrations. Both genotypes were similarly tolerant to amiloride exposure for 4 days and developed similar sodium retention and body weight gain after induction of nephrotic syndrome. Proteolytic processing of γENaC leading to increased expression of distally cleaved γENaC at ~54 kDa was stimulated in both γENaC^wt/wt^ and γENaC^ki/ki^ mice under all interventions without an appreciable difference in the migration pattern.

**Conclusion:**

Mice harboring the RKRK186QQQQ mutation of the distal polybasic tract develop hyperaldosteronism under a low sodium diet, pointing to the relevance of this tract for sodium preservation. However, proteolytical processing of γENaC in these mice appears to be compensated for by the involvement of other adjacent cleavage sites.

## 1 Introduction

The epithelial sodium channel (ENaC) expressed in the distal nephron is a heterotrimer consisting of an α‐, β‐, and γ‐subunit, each encoded by different genes. ENaC plays a decisive role in sodium and potassium homeostasis. This becomes most evident in adult mice with inducible deletion of ENaC which develop severe and eventually lethal sodium wasting and hyperkalemia [1]. Among many factors regulating ENaC‐mediated sodium transport, channel activation through proteolytic processing by serine proteases is a specific feature of ENaC [2, 3]. Proteolytic cleavage takes place at specific sites within the extracellular loops of the α‐ and γ‐subunit but not the β‐subunit and releases inhibitory tracts. This probably causes a conformational change of the channel favoring its open state [3, 4]. During intracellular channel maturation cleavage occurs at two furin cleavage sites (proximal and distal) in αENaC and one proximal in γENaC. A final cleavage event takes place at the plasma membrane where γENaC is cleaved by membrane‐bound proteases and/or extracellular proteases in a region distal to the furin site. The open probability of ENaC increases after proximal cleavage of γENaC and reaches almost 100% after distal cleavage [3, 4, 5]. The proximal and distal cleavage sites on γENaC are characterized by polybasic tracts consisting of the sequence 143RKRR and 186RKRK of murine γENaC. The latter has been referred to as prostasin cleavage site to indicate the preferred action of the membrane‐anchored serine protease prostasin on channel activation [6]. In vitro, proteolytic processing of γENaC has been found to have a dominant role in channel activation as removal of the inhibitory tract from the γ‐subunit in the absence of α‐subunit cleavage resulted in nearly full activation of the channel [5].

In vivo, proteolytic processing of γENaC has been found to be stimulated in mice treated with a low sodium or high potassium diet, pharmacologic ENaC blockade or treatment with aldosterone [7, 8]. Induction of experimental nephrotic syndrome similarly led to increased proteolytic processing of γENaC [7]. In the latter study [7], refinement of the Western blot technique allowed to discriminate between γENaC cleaved at the proximal (furin, ~57–60 kDa) and distal site (~55 kDa) using an antibody directed against the intracellular C‐terminus. All of the abovementioned maneuvers significantly increased the expression of distally cleaved γENaC, suggesting the relevance of processing at the distal site for the stimulation of ENaC activity in vivo. So far, the physiologically relevant proteases involved in proteolytic ENaC activation remain to be identified. From in vivo studies in mice, prostasin, TMPRSS2 (transmembrane serine protease 2) and kallikrein‐1 were shown to interfere with proteolytic processing of γENaC [9, 10, 11, 12].

To study the impact of proteolytic processing of ENaC on sodium homeostasis in vivo, mice carrying mutations of the furin cleavage sites in α‐ and γENaC have been generated [13, 14]. The authors found that mice developed compensatory hyperaldosteronism under a low sodium diet whereas sodium homeostasis was not affected in mice carrying a mutation of the proximal furin cleavage site in γENaC (RKRR143QQQQ). In the latter, the inhibitory tract remained attached as the proximal cleavage product at ~57 kDa was not detectable [13]. So far, mice carrying a mutation of the distal polybasic tract of γENaC (186RKRK) have not been studied. This site has been shown to be of relevance for prostasin under physiologic conditions [6] and possibly for aberrantly proteases filtered in nephrotic syndrome as urine samples from nephrotic humans and mice were found to contain proteolytic activity against the distal polybasic tract [15]. In this study, we generated mice carrying a mutation of the distal polybasic tract of γENaC (RKRK186QQQQ) and phenotyped them for ENaC‐mediated homeostasis under various maneuvers that are known to induce proteolytic processing of γENaC.

## 2 Materials and Methods

### 2.1 Superovulation and Housing of Mice

C57BL/6NRj mice (Janier Labs) were superovulated at the age of 3–4 weeks with 5 IU of PMSG (PMSG‐Intervet, MSD, Hölzel‐Diagnostika, Germany) at 2:00 pm and 5 IU HCG (Ovogest, MSD, Germany) 48 h later. Superovulated mice were crossed overnight with C57BL/6NRj males. Zygotes were prepared from plug‐positive females around 9:00 am and stored until use in EmbryoMax Enhanced KSOM solution (MR‐101, Sigma‐Aldrich) at 37°C in 5% CO2. Animals were housed on low‐dust aspen granulate under standard conditions (55% ± 10% relative humidity and 22°C ± 2°C) in open cages with a 12/12‐h dark/light cycle. They had ad libitum access to tap water and a 25 kGy irradiated breeding and maintenance diet (1318 and 1328, Altromin, Germany). Cages contained additional nesting material and mouse huts as enrichment.

### 2.2 Generation of Knock‐In‐Mice Carrying a Mutation of the Distal Polybasic Tract of the γENaC Gene

The substitution of the four amino acid sequence RKRK with a stretch of 4 glutamine residues (QQQQ) in the γENaC loci (*Scnn1g* gene; MGI:104695) was performed by CRISPR/Cas9 technology. The sgRNA (5′‐ACT TCT TCA CTG GTG GGA AG‐3′) was selected with the help of the Alt‐R CRISPR HDR Design Tool from IDT. Dissolving of sgRNA (IDT) was performed in IDTE‐ buffer (IDT, Coralville, USA) at a concentration of 50 μM and stored at –20°C until use. A 140 nucleotides single stranded oligonucleotide (ssODN) (5′‐CCTGATCCCATTGTTGGTCTTCAATGAGAACGAGAAGGGAAAGGCCAGGGACTTCTTCACTGGTCAGCAGCAGCAGATCAGTGGGAAAATCATACACAAGGCTTCTAATGTCATGCACGTTCATGAGTCGAAGAAAC‐3′, mutated sequence underlined) was used as template for homologous recombination. The oligo was HPLC purified and controlled by mass spectroscopy (Metabion, Planegg, Germany) and dissolved before use in IDTE buffer.

Electroporation was performed according to the protocol from Tröder et al. [16]. Briefly, 4 μM sgRNA and 4 μM Cas9 protein (ESPCAS9PRO, Sigma‐Aldrich) were mixed in a final volume of 18 μL Opti‐MEM (Thermo Fisher Scientific, 31985062), premixed and incubated at room temperature for 10 min. After brief centrifugation, 2 μL ssODN (f.c. 10 μM) were added. The RNP‐DNA solution was stored on ice until use. Electroporation of up to 50 mouse zygotes with 20 μL RNP‐DNA solution was performed in 1 mm cuvettes with a square‐wave double pulse (30 V for 3 ms with a gap of 100 ms) in a BTX ECM830 electroporator (Harvard Apparatus, USA). After electroporation zygotes were washed 5 times in M16 medium (MR‐016, Sigma‐Aldrich) and stored finally in EmbryoMax Enhanced KSOM medium (MR‐101, Sigma‐Aldrich). Embryos were cultured overnight at 37°C in 5% CO_2_ and transferred as 2‐cell embryos with a surgical embryo transfer in the oviducts of pseudopregnant foster mice (Swiss, Janvier Labs, France). Transgenic offspring was identified by genotyping. Heterozygous knock‐in mice were backcrossed to the background 129S1/SvImJ for at least 6 generations. All genotypes were born at the expected Mendelian frequency.

#### 2.2.1 Genotyping Protocol

Genotyping was done by polymerase chain reaction (PCR) from ear tissues collected from the offspring at 3 weeks of age. After tissue lysis, digestion and extraction of genomic DNA using a commercial kit (EchoLUTION Tissue DNA Micro Kit, AIM GmbH, Germany), gDNA was purified after treatment with RNAse and a gDNA‐binding column. PCR was performed using the primers listed in Table S1. After denaturation for 5 min at 94°C, annealing was induced for 30 s at 68°C. Amplification was done at 72°C for 45 s over 35 cycles. Final extension was done for 7 min at 72°C. The PCR products were separated using agarose gel electrophoresis. The gel was stained by Nucleic Acid Gel Stain solution (GelRed, Biotium, USA), and results were recorded under a gel‐imaging system (Bio Rad, USA). Because the amplicons of the wild‐type and mutated allele had the same size, two separate PCR runs had to be performed to determine the correct genotype. A representative image of the obtained results is shown in Figure S1.

#### 2.2.2 Mouse Studies

Experiments were performed on 3–6‐month‐old RKRK186QQQQ or γENaC^ki/ki^ mice and their wild‐type littermates, with mice of both sexes (Table S2). Mice were kept on a 12:12‐h light–dark cycle and fed a standard diet (ssniff, sodium content 0.24% corresponding to 104 μmol/g, potassium content 1.03%, corresponding to 263 μmol/g, Soest, Germany) with tap water ad libitum (measured sodium concentration 1 mM). The ambient temperature was kept between 22°C and 24°C as per official regulations. Sodium balance was studied in metabolic cages for 1 day under a control diet (C1000, Altromin, Lage, Germany, sodium content 106 μmol/g) and 4 days under a low sodium diet (C1036, Altromin, Germany, sodium content 7 μmol/g). ENaC activity was estimated from the amiloride‐sensitive natriuresis under control diet and on the 4th day under a low sodium diet. To this end, mice were injected with vehicle (5 μL/g body weight [bw] injectable water) and amiloride (10 μg/g bw; Sigma) on the next day to determine urinary sodium excretion during 6 h after injection. This dose was found to result in urinary concentrations sufficient for full inhibition of ENaC over 12 h [17]. Amiloride was dissolved in injectable water at a concentration of 2 mg/mL. The solution was freshly prepared and used the same day or stored at 4°C for the 4 day treatment described in the next sentence. To study the effect of a prolonged ENaC blockade mice were treated in metabolic cages with amiloride injected subcutaneously twice daily over 4 days (10 μg/g bw). Experimental nephrotic syndrome was induced by intravenous injection of doxorubicin into the retrobulbar plexus (14 μg/g bw, Doxo‐cell, stock 2 mg/mL) [18, 19, 20]. Daily body weight, food and fluid intake were monitored by weighing the food pellets and the water bottles. Spot urine samples were collected in the morning by bladder massage [18]. 180 μL of blood was collected in heparinized capillaries (Hirschmann, Eberstadt, Germany) from untreated mice under control conditions after puncture of the retrobulbar plexus using a 10 μL capillary under anesthesia with 5% isoflurane. Blood was also collected at sacrifice on day 4 in mice subjected to a low sodium diet and amiloride injections and on day 10 in mice subjected to experimental nephrotic syndrome. Plasma was stored at −20°C until analysis.

All mouse experiments were conducted according to the National Institutes of Health Guide for the Care and Use of Laboratory Animals and the German law for the welfare of animals and were approved by local authorities (Regierungspraesidium Tuebingen).

#### 2.2.3 Laboratory Measurements

Urinary creatinine was measured with a colorimetric Jaffé assay (Labor+Technik, Berlin, Germany), urinary sodium and potassium concentration with flame photometry (Eppendorf EFUX 5057, Hamburg, Germany). 24 h urinary sodium and potassium excretion was normalized to urinary creatinine concentration and food intake. Plasma urea was measured enzymatically using a colorimetric assay (Labor+Technik, Berlin, Germany). Plasma sodium and potassium were measured using an IL GEM Premier 3000 blood gas analyzer (Instrumentation Laboratory, Munich, Germany). Plasma aldosterone and corticosterone concentrations were determined using an EIA (IBL, Hamburg, Germany). Protein concentrations from urine or kidney lysates were determined using the Bradford method (Bio‐Rad, Hercules, USA).

#### 2.2.4 Western Blot Analyses

Western blot analysis of ENaC subunits was performed from a membrane protein preparation of kidney cortex. Half of the snap‐frozen kidney per mouse was sliced, and the cortex was dissected using a scalpel. Homogenization was performed using a Dounce homogenizator in 1 mL lysis buffer containing 250 mM sucrose, 10 mM triethanolamine HCl, 1.6 mM ethanolamine and 0.5 EDTA at pH 7.4 (all Sigma) [21]. During all preparation steps, aprotinin (40 μg/mL) and a protease inhibitor cocktail (final concentration 0.1 x stock; mini‐complete, Roche) were present to avoid ENaC cleavage in vitro. Homogenates were centrifuged at 1000 g for removal of the nuclei. Subsequently, the supernatant was centrifuged at 20000 g for 30 min at 4°C, and the resulting pellet containing plasma membranes was resuspended and diluted to a concentration of 5 mg/L. Native samples were boiled in Laemmli buffer at 70°C for 10 min (α‐ and βENaC). For analysis of γENaC and its cleavage products, samples were deglycosylated using PNGaseF according to the manufacturer's instructions (NEB, Ipswich, USA) [7, 22]. First, samples were denatured with a glycoprotein denaturing buffer. Samples were then incubated with glycobuffer, NP‐40 and PNGaseF for 1 h at 37°C. Subsequently, 20 μg of sample was loaded on an 8% (γENaC) or 4%–15% (α‐ and βENaC) polyacrylamide gel for electrophoresis. After transfer to nitrocellulose membranes (Amersham GE healthcare), the blocked blots were incubated with the primary antibodies. Signals were detected using fluorescent secondary antibodies labeled with IRDye 800CW or IRDye 680RD and a fluorescence scanner (Licor Odyssey, Lincoln, USA). For loading control, total protein was measured using Revert Total Protein Stain (Licor, Lincoln, USA). Primary antibodies against α‐ and βENaC subunits were raised against mouse orthologues of the immunogenic peptides reported by Masilamani et al. for rats [23]. Anti‐αENaC was generated by Pineda antibody service and a kind gift of Prof. Christoph Korbmacher (University of Erlangen). Anti‐βENaC was raised by Proteogenix. Anti‐γENaC directed against the sequence of rat was purchased from Stressmarq (SPC‐405). Anti α‐, β‐ and γENaC were diluted 1:1000 each. The linear range of the signal was established (Figure S2).

#### 2.2.5 Immunohistochemistry

For analysis of tissue expression of γENaC, perfusion‐fixed kidneys were collected under control conditions or at the end of each treatment. To this end, mice were anesthetized with xylazine (8 μg/g BW, stock 20 mg/mL) and ketamine (100 μg/g BW, stock 50 mg/mL) given intraperitoneally. The abdomen was opened and the aorta cannulated with an 18‐gauge venous catheter. Subsequently, mice were perfused with each 20 mL of 0.9% NaCl and 4% formalin using an infusion pump. Paraffin‐embedded formalin‐fixed sections (1 μm) were deparaffinized with ethanol and rehydrated using standard protocols. Antigen retrieval was accomplished after heating for 5 min in antigen retrieval solution pH 6.1 (DAKO Deutschland GmbH, Hamburg, Germany) using a pressure cooker (Rommelsbacher, Germany). Kidney sections were blocked with avidin and biotin for each 15 min, followed by blocking for another 30 min with normal goat serum diluted 1:5 in 50 mM tris(hydroxymethyl)‐aminomethane (Tris), pH 7.6 and 0.1 mL Tween 20%, supplemented with 5% (w/v) skim milk (Bio‐Rad Laboratories, Munich, Germany). Sections were incubated overnight at 4°C with the primary antibodies (dilution 1:250 for Anti‐γENaC) and subsequent washing in Tris buffer (50 mM) Tris, pH 7.4, supplemented with 0.05% (v/v) Tween 20 (Sigma‐Aldrich, Munich, Germany; 3 x). A secondary antibody (biotinylated goat anti‐rabbit, Vector Laboratories, Burlingame, CA, USA; 1:500) was applied for 30 min at room temperature. Sections were further processed using the VectaStain ABC kit according to the manufacturer's instructions and DABImmPact (both Vector Laboratories) as substrate. Finally, the sections were counterstained in hematoxylin, dehydrated, and mounted for observation using a Zeiss upright microscope. For each staining, 4 sections from at least two mice were analyzed at 20× and 63× magnification in order to be able to make a qualitative statement.

#### 2.2.6 Statistical Analysis

Sample size was estimated to detect differences of 1.0, 1.25, and 1.5 fold of the standard deviation of the primary end points 24 h sodium excretion, density of distally cleaved γENaC, and body weight gain after induction of experimental nephrotic syndrome, respectively. This yielded a sample size between 9 to 17 mice per genotype at an alpha‐ and beta‐level of 0.05 and 0.80, respectively. Data are provided as means with SEM. Data were tested for normality with the Kolmogorov–Smirnov‐Test, D'Agostino and Pearson omnibus normality test, and Shapiro–Wilk‐Test. Variances were tested using the Bartlett's test for equal variances. Accordingly, data were tested for significance with parametric ANOVA followed by Dunnett's Multiple Comparison post‐test or nonparametric Kruskal–Wallis followed by Dunn's Multiple Comparison post‐test, paired or unpaired Student's *t*‐test, or Wilcoxon‐test where applicable using GraphPad Prism 10 (GraphPad software, San Diego, USA). Densitometric analysis of the Western blots was done using Image Studio Version 3.1.4 and Empiria Studio Version 1.3.0.83 (Licor). A *p* value < 0.05 at two‐tailed testing was considered statistically significant.

## 3 Results

### 3.1 Amiloride‐Sensitive Natriuresis and Responses to a Low Sodium Diet as Well as to a 4‐Day Treatment With Amiloride

**FIGURE 1:**
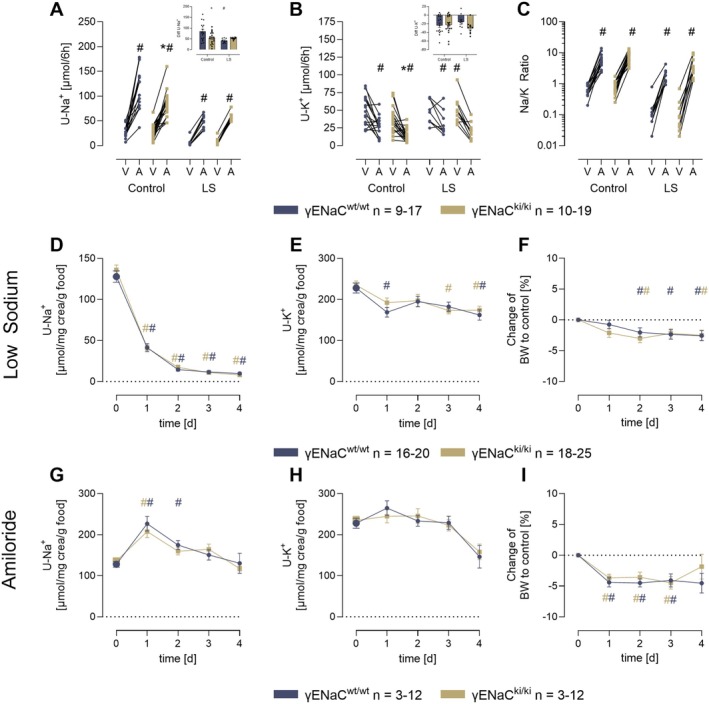
Responses to a bolus injection of amiloride, a low sodium diet as well as to a 4‐day treatment with amiloride. Urinary sodium (A) and potassium (B) excretion as well as urinary Na/K ratio (C) after injection of vehicle (V) and amiloride (10 μg/g bw, A) under control conditions and on the 4th day under a low sodium (LS) diet. Urine was collected over 6 h in metabolic cages without access to drink and food. Course of urinary sodium (D) and potassium excretion (E) as well as changes in body weight (F) under a low sodium diet. Values of the 24 h urine were normalized to urinary creatinine concentrations and food intake to correct for occult urine losses and differences in food intake. Course of urinary sodium (G) and potassium excretion (H) as well as changes in body weight (BW) (I) under a 4 day treatment with amiloride given subcutaneously twice daily (2 × 10 μg/g bw). ^#^significant difference (*p* < 0.05) between control and intervention in mice of the same genotype (ANOVA with Dunnetts multiple comparison test or Kruskal–Wallis with Dunn's multiple comparison test) * significant difference (*p* < 0.05) between genotypes (two‐way ANOVA and/or unpaired *t*‐test or Wilcoxon test) Note that the control values (d,e,g,h) at day 0 were pooled from all experimental series (indicated by larger symbols).

To test ENaC activity in vivo, amiloride was injected as a bolus and urine was collected over the next 6 h. As shown in Figure 1A, the natriuretic response was slightly but significantly lower in γENaC^ki/ki^ mice compared to γENaC^wt/wt^ mice under a control diet (114 ± 11 vs. 83 ± 6 μmol/6 h, *p* < 0.05). The same was true for the antikaliuretic action of amiloride (27 ± 4 vs. 13 ± 2 μmol/6 h, *p* < 0.05, Figure 1B). The urinary Na/K ratio calculated from the urinary concentrations was, not different between the genotypes (Figure 1C). Under control conditions, food and fluid intake as well as urine output was identical in both genotypes and increased under a low sodium diet (Figure S3A–C). Urinary sodium excretion was reduced in both genotypes which was paralleled by a tendency toward lower urinary potassium excretion (Figure 1D,E). Body weight was reduced to a similar extent and was stable in both genotypes at day 4 (Figure 1F). ENaC activity as assessed by amiloride bolus on the 4th day of the low sodium diet was not significantly different between the genotypes anymore (Figure 1A–C).

To test the counter‐regulation to prolonged ENaC inhibition, amiloride was applied subcutaneously twice daily over 4 days. Food and fluid intake as well as urine output increased during amiloride treatment in both genotypes (Figure S2D–F). Urinary sodium excretion was stimulated on day 1 and returned to normal levels, indicating counter‐regulation and tolerance (Figure 1G). There was no difference between the genotypes. Urinary potassium excretion remained constant during amiloride administration (Figure 1H). Body weight fell significantly in both genotypes and reached a steady state at a slightly lower level than after a low sodium diet (Figure 1I). During low sodium diet and prolonged amiloride treatment, there were no mice experiencing critical body weight loss and deterioration.

### 3.2 Plasma Analytes Under a Low Sodium Diet and Prolonged Amiloride Administration

**FIGURE 2:**
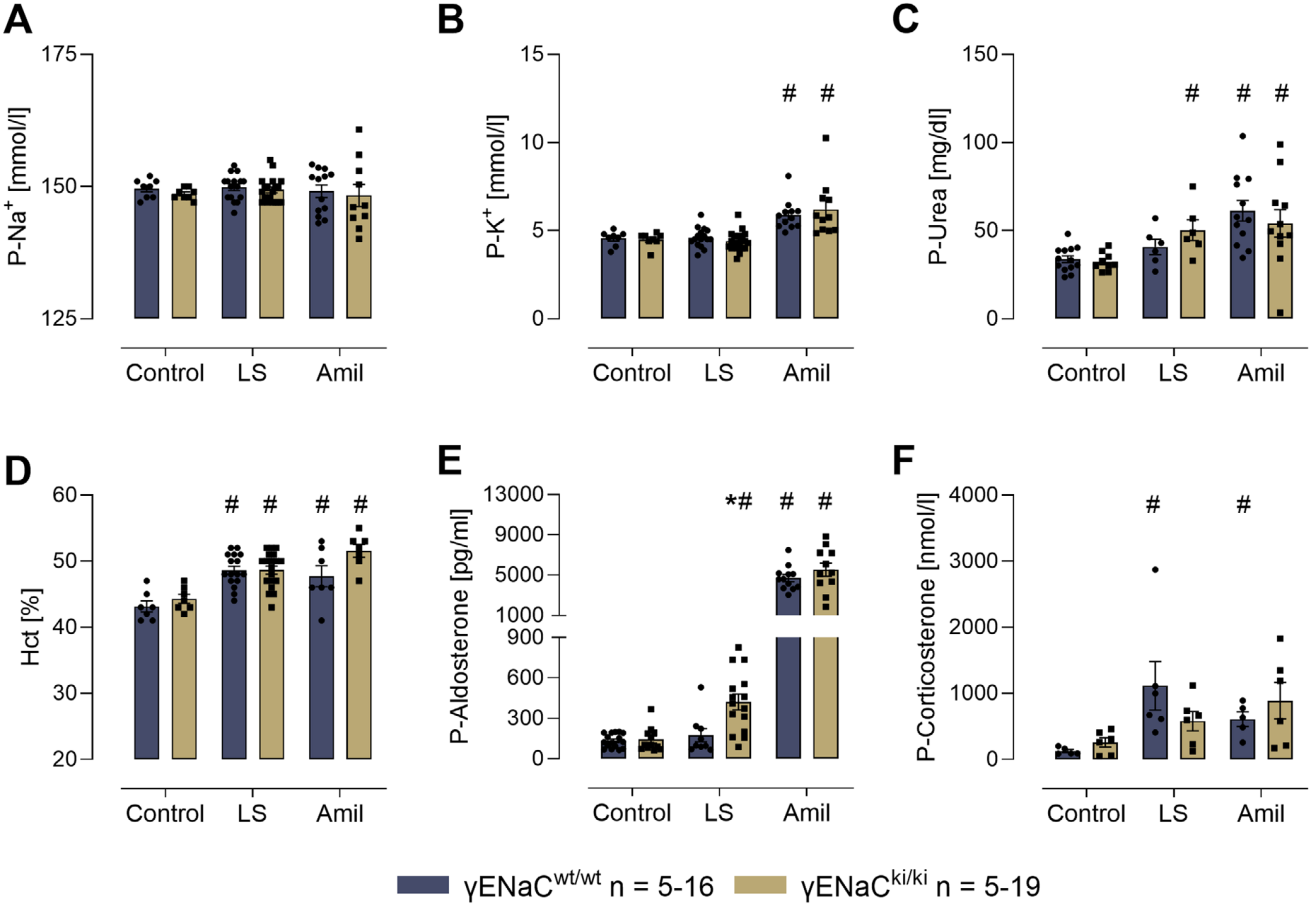
Plasma sodium (A), potassium (B), urea (C), hematocrit (D), aldosterone (E), and corticosterone (F) concentrations before and after a low sodium (LS) diet and a 4‐day treatment with amiloride (Amil). ^#^significant difference (*p* < 0.05) between control and intervention in mice of the same genotype (ANOVA with Dunnetts multiple comparison test or Kruskal–Wallis with Dunn's multiple comparison test) * significant difference (*p* < 0.05) between genotypes (unpaired *t*‐test or Wilcoxon test).

Under control conditions, there were no significant differences of the plasma sodium, potassium urea, aldosterone and corticosterone concentrations as well as the hematocrit between the genotypes (Figure 2A–F). Under both maneuvers, plasma sodium concentrations remained constant (Figure 2A), whereas plasma potassium concentrations were elevated under a prolonged amiloride administration in both genotypes (Figure 2B). Under a low sodium diet, plasma urea concentrations significantly increased in γENaC^ki/ki^ mice whereas under prolonged amiloride administration they were significantly increased in both genotypes (Figure 2C). Hematocrit increased in both genotypes under a low sodium diet and to a larger extent under prolonged amiloride treatment (Figure 2D). Under a low sodium diet, plasma aldosterone concentrations significantly increased in γENaC^ki/ki^ mice, reaching significantly higher values than in γENaC^wt/wt^ mice (Figure 2E). However, under amiloride treatment plasma aldosterone strongly increased in both genotypes to the same extent (Figure 2E). Plasma corticosterone concentrations significantly increased in γENaC^wt/wt^ mice under a low sodium diet and amiloride treatment, this increase was similar in γENaC^ki/ki^ mice, however, did not reach statistical significance (Figure 2F).

### 3.3 Induction of Experimental Nephrotic Syndrome and Sodium Retention

**FIGURE 3:**
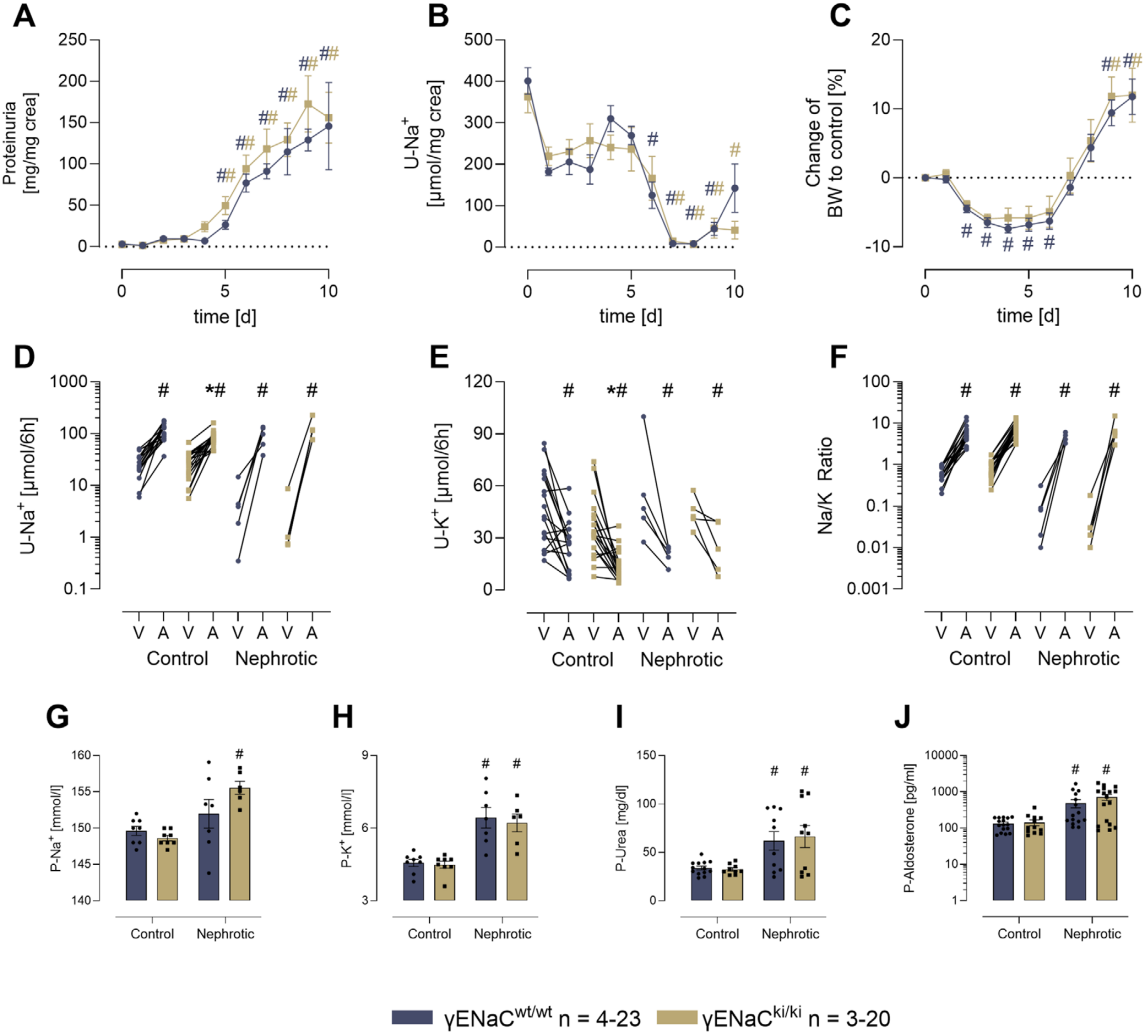
Induction of experimental nephrotic syndrome and sodium retention. Course of urinary protein excretion (A), urinary sodium excretion in spot urine samples (B), and changes of body weight (C) after induction of nephrotic syndrome. Urinary sodium (D) and potassium excretion (E) as well as urinary Na/K ratio (F) after acute administration of the ENaC inhibitor amiloride (A, 10 μg/g) or vehicle injection (V, injectable water, 5 μL/g). Values were normalized to urinary creatinine concentrations to correct for dilution. Plasma sodium (G), potassium (H), urea (I), and aldosterone concentrations (J) under control conditions and 10 days after induction of nephrotic syndrome. ^#^significant difference (*p* < 0.05) between control and intervention in mice of the same genotype (ANOVA with Dunnetts multiple comparison test or Kruskal–Wallis with Dunn's multiple comparison test) * significant difference (*p* < 0.05) between genotypes (two‐way ANOVA and/or unpaired *t*‐test or Wilcoxon test).

To test the relevance of the distal polybasic tract for sodium retention in experimental nephrotic syndrome, mice were injected with doxorubicin at day 0 to induce nephrotic proteinuria. As shown in Figure 3A, both genotypes developed proteinuria to the same extent, exceeding the threshold of 100 mg/mg creatinine at day 6 for triggering sodium retention as established previously [19, 24]. After doxorubicin injection, food and fluid intake dropped in γENaC^ki/ki^ mice (Figure S4A–B). Despite almost constant sodium intake (Figure S3C), urinary sodium excretion decreased in both genotypes and approached values near to zero at day 7–8 in both genotypes (Figure 3B). After transient body weight loss following doxorubicin injection, body weight increased in both genotypes and was accompanied by development of ascites (Figure 3C).

Urinary potassium excretion was reduced in the first three days after doxorubicin injection in both genotypes, then recovered and increased after 5 days despite constant intake (Figure S4D,E). ENaC activity as estimated from the response to amiloride was stimulated in both genotypes under nephrotic conditions (Figure 3D–F). Plasma sodium concentrations increased in nephrotic γENaC^ki/ki^ mice (Figure 3G), whereas plasma potassium and urea concentrations increased in nephrotic mice of both genotypes, suggesting reduced renal function (Figure 3H,I). Both genotypes developed similar degree of hyperaldosteronism after induction of nephrotic syndrome (Figure 3J), which is a known feature of this model [19, 25]. Plasma corticosterone concentrations tended to increase in both genotypes to a similar extent without reaching statistical significance (Figure S4F).

### 3.4 Localization of γENaC in the Kidney

**FIGURE 4:**
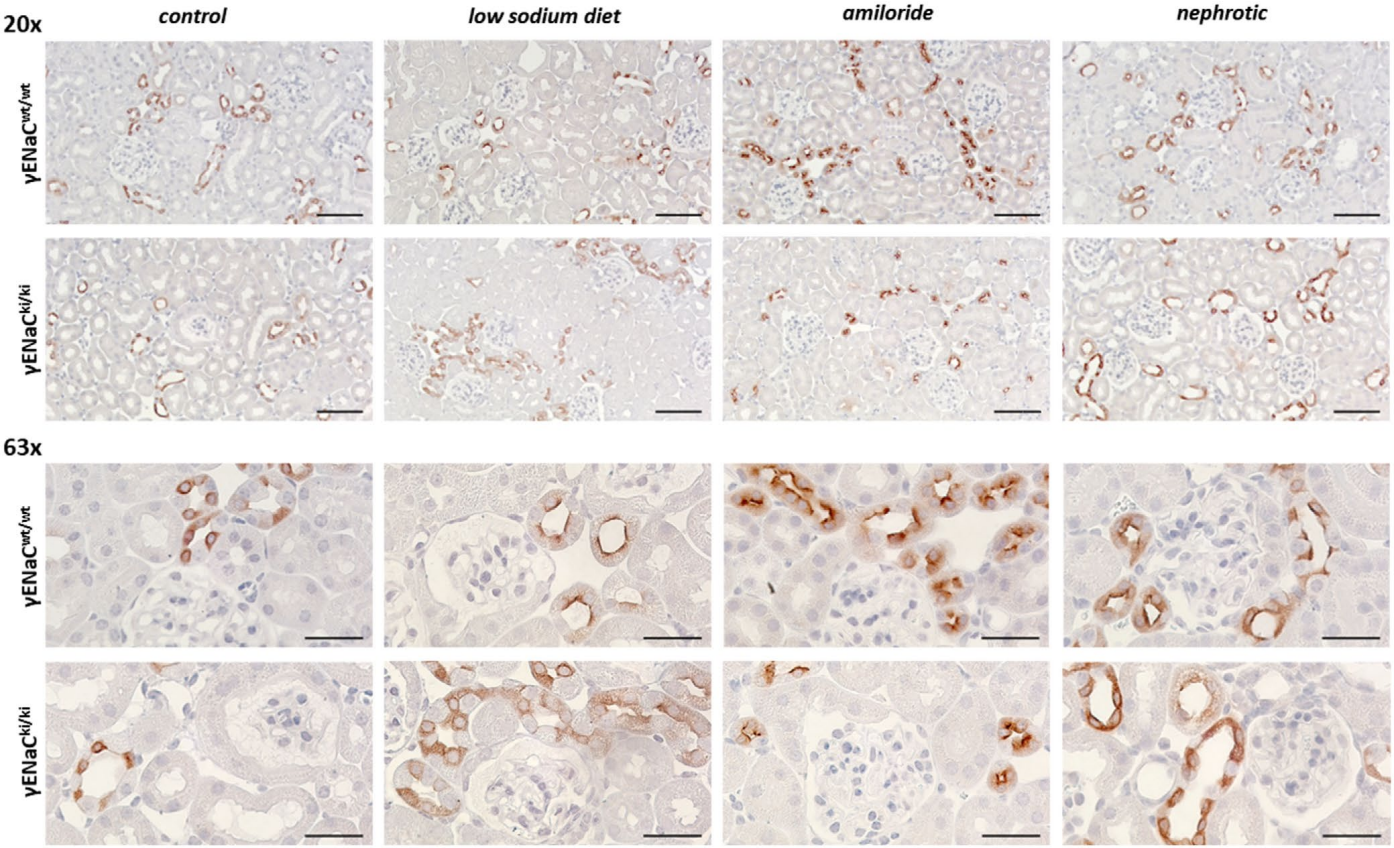
Tissue expression of γENaC under control conditions and after a 4 day treatment with a low sodium diet or amiloride as well as 10 days after induction of nephrotic syndrome. Representative staining of kidney sections stained for γENaC at 20‐fold (upper panel, scale 5 μm) and 63‐fold (lower panel, scale 20 μm) magnification. The antibody was the same as used for Western blot.

We next analyzed the localization of γENaC in the kidney using immunohistochemistry. Under control conditions, γENaC staining was characterized by a predominantly cytosolic pattern in γENaC^wt/wt^ mice without an obvious difference to γENaC^ki/ki^ mice (Figure 4), [11]. After a 4 day treatment with a low sodium diet or amiloride as well as 10 days after induction of nephrotic syndrome, the expression of γENaC shifted to the apical plasma membrane in γENaC^wt/wt^ mice, as previously shown and known as apical targeting [21, 22]. This expression pattern was unaltered in in γENaC^ki/ki^ mice. Therefore, mutation of the distal polybasic tract does not seem to affect trafficking of γENaC.

### 3.5 Expression of ENaC Subunits and Proteolytic Processing in Kidney Lysates

**FIGURE 5:**
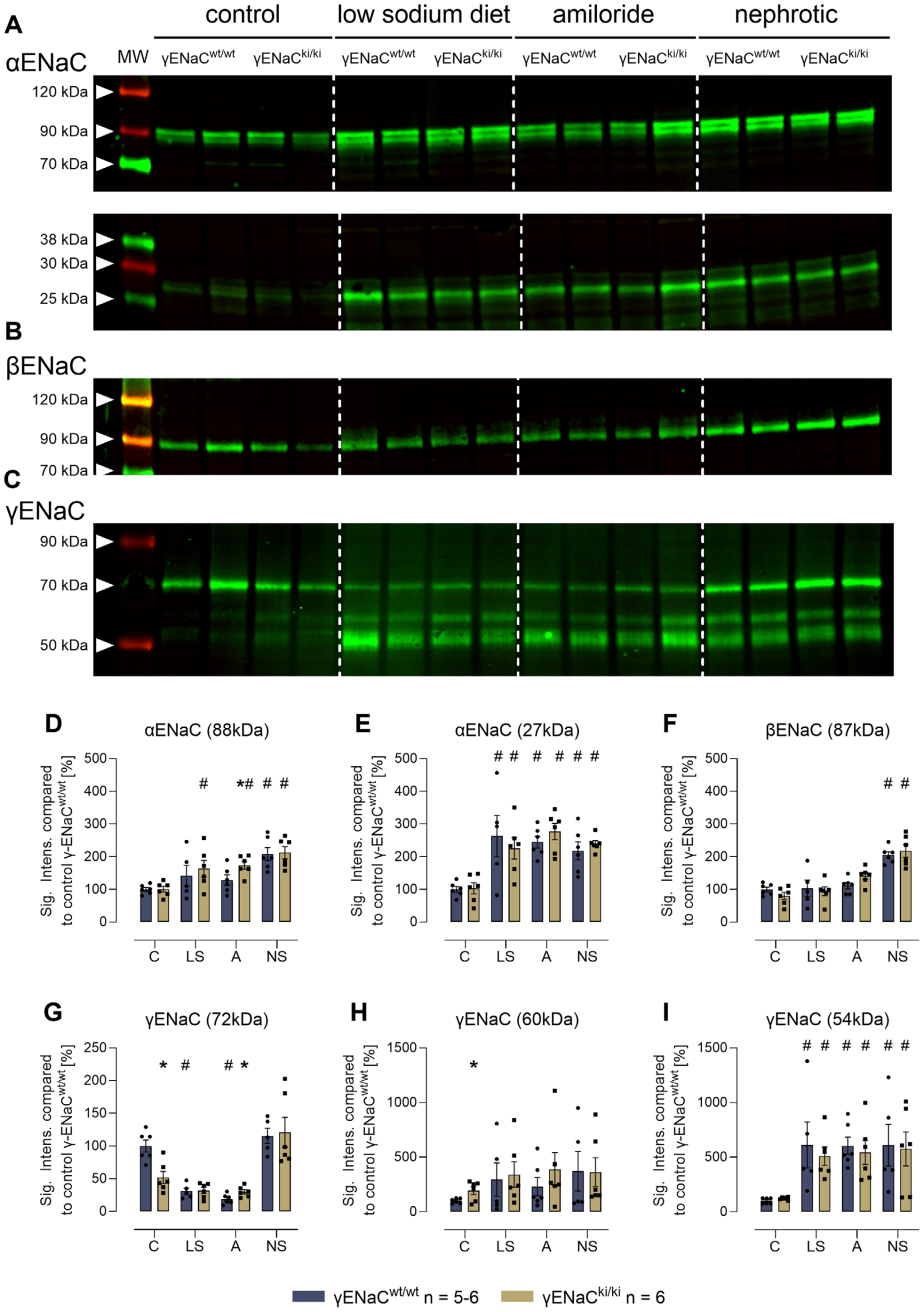
Expression of ENaC subunits and proteolytic processing in kidney lysates under control conditions and after a 4‐day treatment with a low sodium diet or amiloride as well as 10 days after induction of nephrotic syndrome. (A‐C) Representative Western blots showing the expression of α‐, β‐, and γENaC in a plasma membrane preparation of kidney cortex lysates. Note that the samples were deglycosylated before analyzing expression of γENaC and its cleavage products [22]. The white line is only for optical discrimination; it is one blot each, no vertical cutting. (D‐I) Densitometry of the obtained bands normalized for total protein content of each lane. ^#^significant difference (*p* < 0.05) between control and intervention in mice of the same genotype (ANOVA with Dunnetts multiple comparison test or Kruskal–Wallis with Dunn's multiple comparison test) * significant difference (*p* < 0.05) between genotypes (unpaired *t*‐test or Wilcoxon test).

We then studied the expression and proteolytic processing of ENaC subunits using Western blot. In kidney cortex lysates from γENaC^wt/wt^ mice under control conditions, Western blot analyses identified two bands for αENaC at 88 and 27 kDa corresponding to full‐length and a cleavage product after distal cleavage (designated from the N‐terminus; Figure 5A). For βENaC, there was only a single band at 87 kDa corresponding to the full‐length subunit which is not proteolytically processed (Figure 5B). For γENaC there were three bands in deglycosylated samples at 72, 60, and 54 kDa (Figure 5C) corresponding to full‐length, proximally and distally cleaved fragments, respectively [7, 22]. In γENaC^ki/ki^ mice under control conditions, there were no significant differences in the expression of α‐ and βENaC (Figure 5D–F), however, the expression of full‐length γENaC was significantly lower (Figure 5G) and that of proximally cleaved γENaC was significantly increased (Figure 5H). The expression of distally cleaved γENaC was not different in γENaC^ki/ki^ mice (Figure 5I). Under a low sodium diet and prolonged amiloride treatment, expression of full‐length αENaC was stimulated in γENaC^ki/ki^ mice (Figure 5D) whereas the expression of cleaved αENaC was stimulated in both genotypes (Figure 5E). In nephrotic mice of both genotypes, both full‐length and cleaved αENaC was stimulated (Figure 5D,E). The expression of βENaC was stimulated in nephrotic mice of both genotypes, but not under a low sodium diet and prolonged amiloride treatment (Figure 5F).

The expression of full‐length γENaC was reduced in γENaC^wt/wt^ mice under a low sodium diet and prolonged amiloride treatment and remained unaltered under nephrotic conditions (Figure 5G). The expression pattern of full‐length γENaC was similar in γENaC^ki/ki^ mice. Proximally cleaved γENaC at 60 kDa was not affected by the maneuvers in mice of both genotypes (Figure 5H). Regarding the expression of distally cleaved γENaC at 54 kDa, all maneuvers induced a robust and strong stimulation of the expression of this cleavage product in γENaC^wt/wt^ mice, indicating heavy proteolytic processing under a low sodium diet, prolonged amiloride treatment as well as after induction of nephrotic syndrome (Figure 5I). Strikingly, this upregulation was not altered in γENaC^ki/ki^ mice lacking the distal polybasic tract. The corresponding cleavage product migrated at 54 kDa which was not different from the migration pattern seen in γENaC^wt/wt^ mice.

## 4 Discussion

The current study investigated the relevance of the distal polybasic tract of γENaC at 186RKRK for sodium homeostasis in vivo. Using knockin mice carrying a mutation of this site to RKRK186QQQQ (γENaC^ki/ki^), we found that these mice were viable and fertile without any impairment. In maneuvers designed to challenge ENaC function and stimulate proteolytic processing of γENaC, we found that γENaC^ki/ki^ mice had reduced amiloride sensitivity under control conditions, suggesting reduced ENaC activity (Figure 1A). Under a low sodium diet these mice adequately upregulated ENaC activity as indicated by amiloride sensitivity (Figure 1A) and reduced urinary sodium excretion, thereby maintaining sodium homeostasis (Figure 1D–F). However, γENaC^ki/ki^ mice required higher aldosterone stimulation to compensate for the loss of the distal polybasic tract (Figure 2C). These findings suggest the involvement of the distal polybasic tract for sodium homeostasis under a low sodium diet in vivo, however, they also indicate that it is not essential for it. This was most clearly seen in the analysis of proteolytic processing by Western Blot. Here, γENaC was similarly processed in γENaC^ki/ki^ mice as compared to γENaC^wt/wt^ mice and the expression of distally cleaved γENaC was not altered (Figure 5). Surprisingly, challenging γENaC^ki/ki^ mice with a prolonged amiloride treatment was not accompanied by a higher aldosterone secretion as compared to γENaC^wt/wt^ mice, however, the treatment also stimulated very high values in the latter. The most striking result was seen when proteolytic processing was analyzed using Western blot with a refined approach involving deglycosylated samples [7, 22]. Here, the results clearly demonstrated a stimulation of proteolytic processing of γENaC, and the expression of distally cleaved γENaC was upregulated in γENaC^ki/ki^ mice to the same degree as observed in γENaC^wt/wt^ mice (Figure 5), excluding an essential role of the distal polybasic tract for maximally stimulating ENaC activity in vivo. From the migration pattern, the cleavage product representing cleavage at the distal region of the inhibitory peptide had the same size in γENaC^ki/ki^ mice. This suggests that adjacent sites must have been recruited for distal cleavage and removal of the inhibitory peptide. Indeed, the distal region harbors several positively charged lysine and arginine residues which can serve as alternative cleavage sites. In a recent study investigating the role of the serine protease TMPRSS2, mutation of the distal polybasic tract was not sufficient for abolishing proteolytic processing and only mutation of all positively charged residues abolished the stimulatory effect of TMPRSS2 in oocytes [26]. These mutations comprised γRKRK178AAAA in combination with K168A, K170A, R172A and K189A as found in the distal region of the human orthologue of γENaC. Remarkably, there were no preferences of TMPRSS2 for any of these sites. It would be interesting to generate and study mice with additional mutations of possible cleavage sites in γENaC, eventually abolishing full proteolytic activation of γENaC.

In vitro, a multitude of serine proteases were identified that could activate ENaC by proteolytic processing including prostasin [6], kallikrein‐1 [12], TMPRSS2 [26], plasmin [27], factor VII‐activating protease [28], plasma kallikrein [29] and others. These serine proteases have in common that they belong to the S1 clan of the trypsin‐like proteases and cleave after positively charged lysine and arginine residues. While doing so, their sequence specificity is limited and many cleave promiscuously. Therefore, the distal region of γENaC harboring several lysine and arginine residues contains redundant cleavage sites for serine proteases to cleave and activate ENaC. This holds true for serine proteases under both physiological conditions and experimental nephrotic syndrome (Figure 3) which is characterized by aberrant filtration of plasma serine proteases with a higher molecular weight [30]. Using a peptide substrate containing the murine distal polybasic tract (residues 180–194, FTGRKRKISGKIIHK), urine samples of nephrotic mice and humans were found to cleave the substrate within the polybasic tract, yielding FTGRKR as the dominant cleavage product [15]. This observation suggested the distal polybasic tract might be a preferential site of proteolytic ENaC activation in nephrotic syndrome and stimulated the generation of the knockin mice of this study.

The results with the γENaC^ki/ki^ mice indicate that the distal polybasic tract is not essential for proteolytic processing and suggests that other adjacent cleavage sites are involved. Under all tested challenges, expression of the distally cleaved γENaC was clearly stimulated (Figure 5). Therefore, these results indicate that proteolytic processing of γENaC is involved in ENaC‐mediated sodium handling in vivo. The role of proteolytic processing of γENaC was questioned by results obtained with mice harboring a mutation of the proximal polybasic tract of γENaC (RKRR143QQQQ) [13]. In Western blot analyses using deglycosylated samples, the expression of proximally cleaved was almost absent, while the expression of distally cleaved γENaC was detectable. Under a low sodium diet, mutant mice adequately lowered urinary sodium excretion without developing hyperaldosteronism. The authors concluded that proteolytic processing of γENaC may play a minor role in vivo. However, one must acknowledge that in these mice the inhibitory tract is expected to remain attached at the proximal furin cleavage site. This possibly preserves its inhibitory effect as liberation of the inhibitory peptide is necessary for channel activation by mediating the opening of the channel's gate through releasing interactions that constrain the finger–thumb domain interface of γENaC [6, 31, 32]. Unfortunately, Western blot analyses of γENaC under a low sodium diet was not reported and it remains unclear whether the expression of distally cleaved γENaC was increased in these mutant mice [13]. An alternative explanation for the phenotype of these mice could be that distal cleavage alone is sufficient to activate the channel in vivo. Our results with mice harboring a mutation of the distal polybasic tract (RKRK186QQQQ) suggests that proteolytic processing at the distal polybasic tract is relevant as these mice require hyperaldosteronism to ensure sodium preservation under a low sodium diet. This phenotype was similarly seen in mice carrying a mutation of the distal furin cleavage site in αENaC (RSAR229QSAQ) [14]. In analogy to our study, the abundance of cleavage product of αENaC at ~30 kDa could be stimulated under a low sodium diet, suggesting the involvement of adjacent cleavage sites in the region of the distal furin cleavage site in αENaC [14].

In conclusion, sodium homeostasis and proteolytical processing of γENaC were preserved in mice carrying a mutation of the distal polybasic tract suggesting the involvement of other adjacent cleavage sites. As with redundancy of serine proteases capable of cleaving γENaC there also appears to be a redundancy of cleavage sites in the region of the distal inhibitory tract.

## Author Contributions

**Thomas Ott:** methodology. **Bernhard N. Bohnert:** writing – review and editing, writing – original draft, conceptualization, formal analysis. **Matthias Wörn:** investigation. **Anja Schork:** writing – original draft, writing – review and editing. **M. Zaher Kalo:** investigation, visualization. **Andreas L. Birkenfeld:** writing – original draft, resources. **Daniel Essigke:** conceptualization, investigation, writing – original draft, visualization, writing – review and editing, data curation, validation. **Ferruh Artunc:** conceptualization, funding acquisition, writing – original draft, supervision, project administration, writing – review and editing, methodology. **Xia‐Qing Li:** investigation.

## Funding

This study was supported by grants from the Deutsche Forschungsgemeinschaft (DFG, German Research Foundation) to DE (project number 493665037, MINT‐Clinician Scientist program of the Medical Faculty Tübingen) and FA (project number 457011590 (AR 1092/2‐2)).

## Conflicts of Interest

The authors declare no conflicts of interest.

## Supporting information


**Table S1:** Used primers.
**Table S2:** Number of mice used for the experiments and sex distribution.
**Figure S1:** PCR results from genotyping of γENaCwt/wt and γENaCki/ki mice.
**Figure S2:** Linear range of the used antibodies.
**Figure S3:** Food and fluid intake as well as urine output under a low sodium diet as well as under a 4‐day treatment with amiloride in γENaCwt/wt and γENaCki/ki mice.
**Figure S4:** Food and fluid intake, calculated sodium intake, urinary potassium excretion, calculated potassium intake and plasma concentrations of corticosterone before and after induction of experimental nephrotic syndrome in γENaCwt/wt and γENaCki/ki mice.

## Data Availability

The data that support the findings of this study are available from the corresponding author upon reasonable request.
