# Supplementary material for: Hyperaldosteronism in Mice Lacking the Distal Polybasic Tract of the γ‐Subunit of the Epithelial Sodium Channel During Sodium Restriction
Source: Acta Physiol (Oxf). 2026 Apr 27;242:e70228. doi: 10.1111/apha.70228 (PMC13112053; doi:10.1111/apha.70228)

**Supplementary material:**

**Supplemental Table 1: Used primers.**

| **gene** | **sense/forward**  **5’→3’ orientation** | **antisense/reverse**  **5’→3’ orientation** | **amplicon** |
| --- | --- | --- | --- |
| γENaC^wt/wt^ | agg gac ttc ttc act ggt cgg aag | aag aag aga ctg tgg gac tac c | 180 bp |
| γENaC^ki/ki^ | agg gac ttc ttc act ggt cag cag |  | 180 bp |

**Supplemental Table 2: Number of mice used for the experiments and sex distribution.**

| mice | Σ [mice] |
| --- | --- |
| total | 159 |
| included | 153 |
| excluded all | 6 |
| excluded due to lack of response (non-nephrotic proteinuria) | 6 |
|  |  |
| *treatment groups* | *Σ [mice]* |
|  |  |
| bolus diuretics and control diet | 36 (17 wt; 19 ki) |
| low sodium diet (after 1 day control diet) | 50 (25 wt; 25 ki) |
| amiloride treatment (after 1 day control treatment) | 24 (12 wt; 12 ki) |
| experimental nephrotic syndrome | 43 (23 wt, 20 ki) |
|  |  |
| *sex* | *Σ [mice]* |
|  |  |
| male | 85 (47 wt; 38 ki) |
| female | 68 (30 wt; 38 ki) |
|  |  |

**Supplemental Figure 1: PCR results from genotyping of γENaC^wt/wt^ and γENaC^ki/ki^ mice.**

To determine the correct genotype, two separate PCR runs were run as the size of the amplicon was identical between the amplicons. Lanes from left to right: 50 bp ladder, lanes 2-9 γENaC^wt/wt^ mice, lane 10 γENaC^ki/ki^ mouse, H_2_O and positive controls.


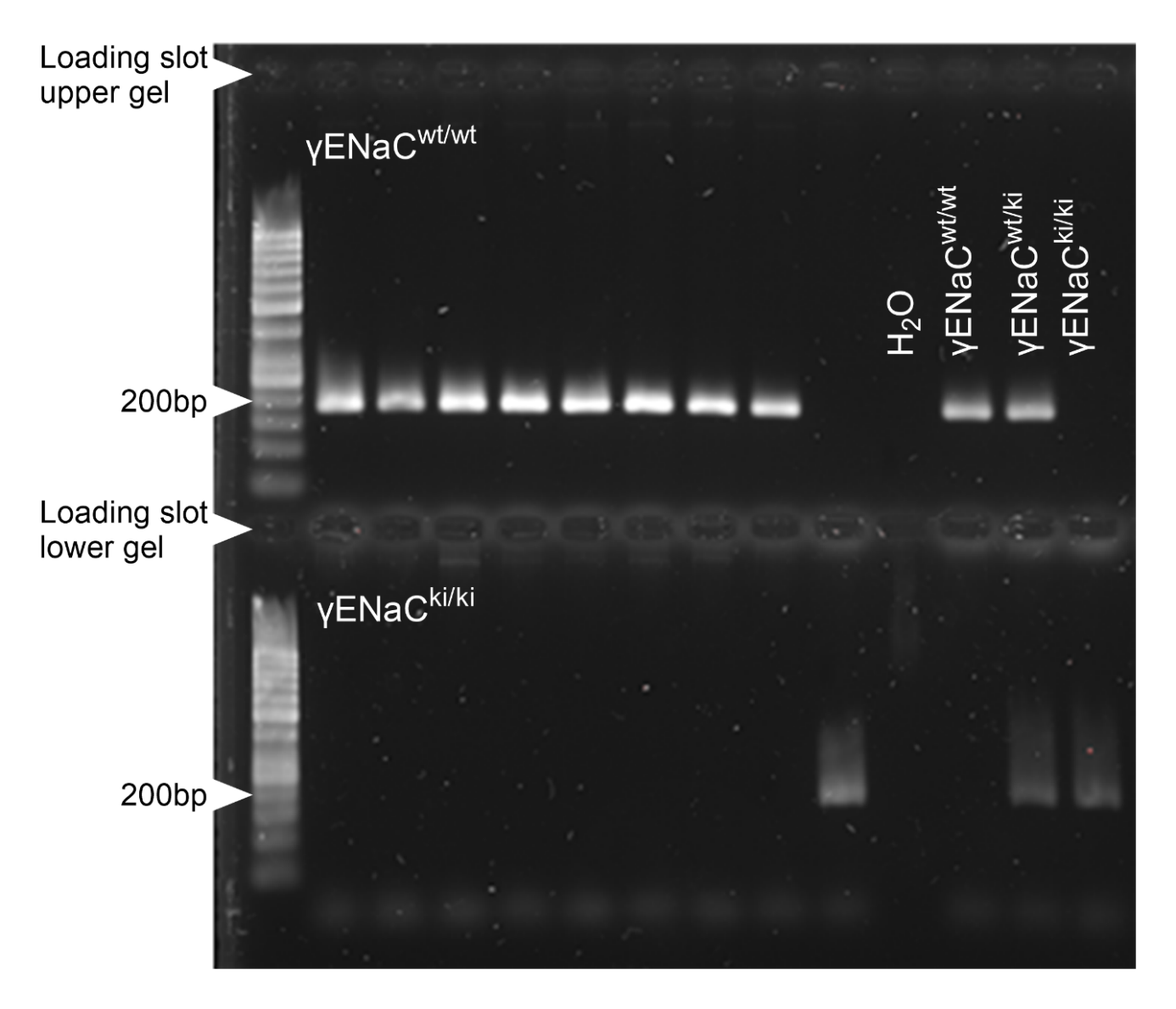


**Supplemental Figure 2: Linear range of the used antibodies**

A Linear range of both the 70 and 26 kDa band detected by Anti-αENaC

| Total protein | Anti-αENaC 70 kDa | Anti-αENaC 26 kDa |
| --- | --- | --- |
| 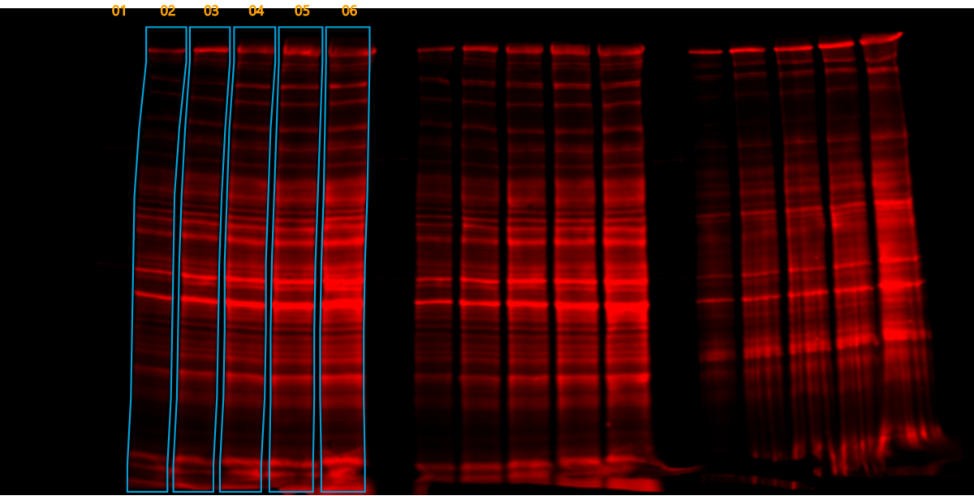 | 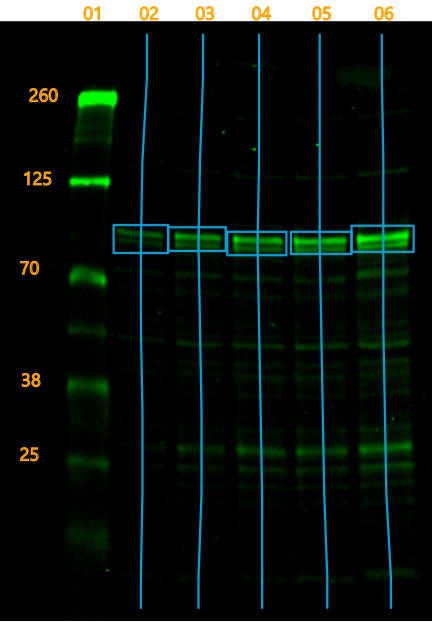 | 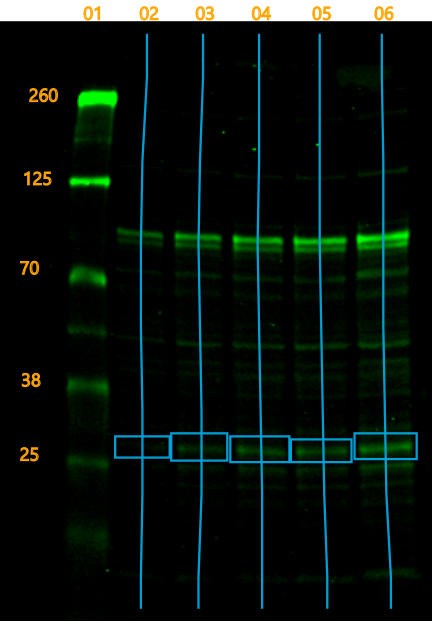 |
| 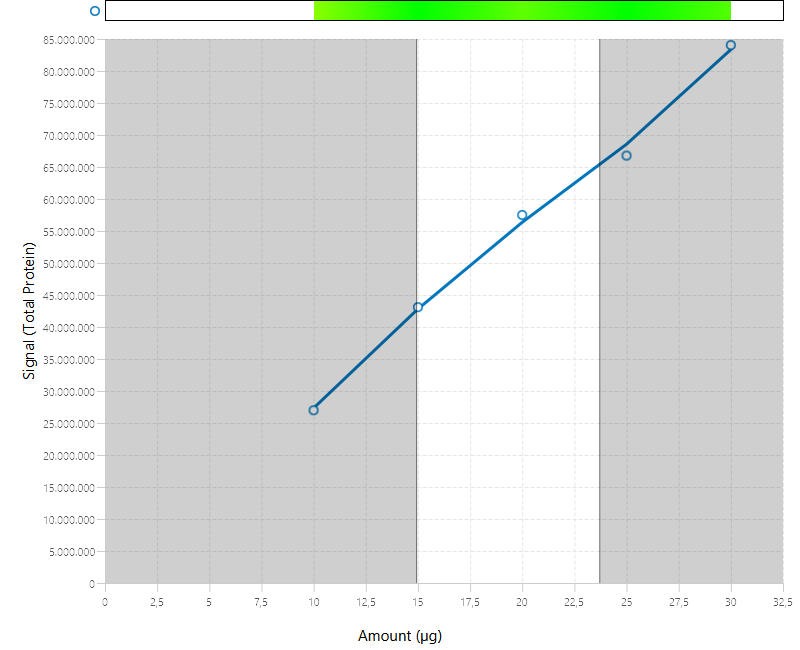 | 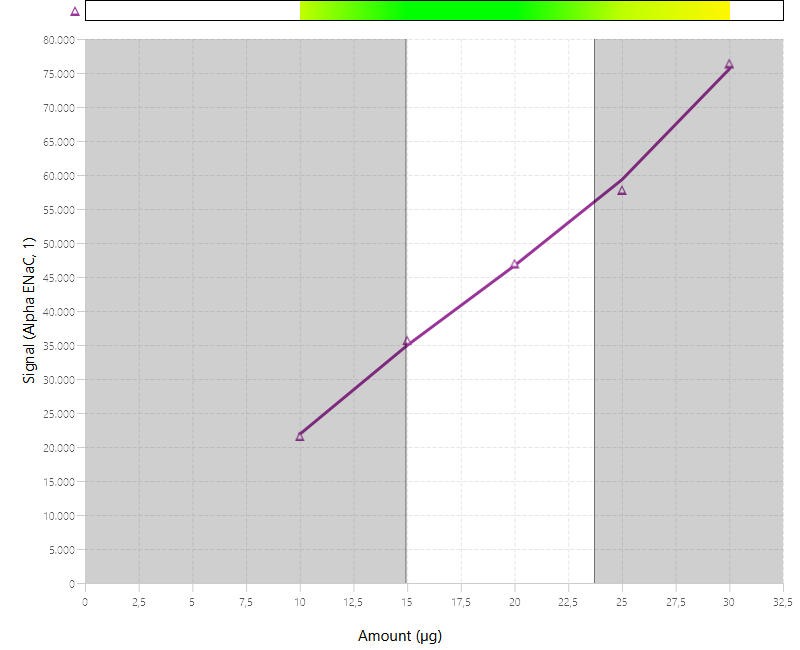 | 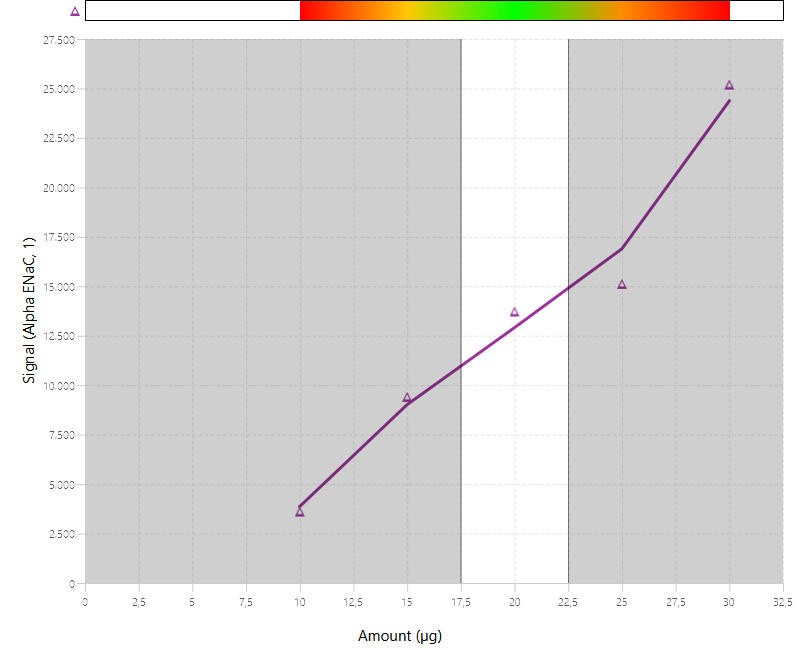 |

**Supplemental Figure 2: Linear range of the used antibodies (continued)**

B Linear range of the 90 band detected by Anti-βENaC

| Total protein | Anti-βENaC |
| --- | --- |
| 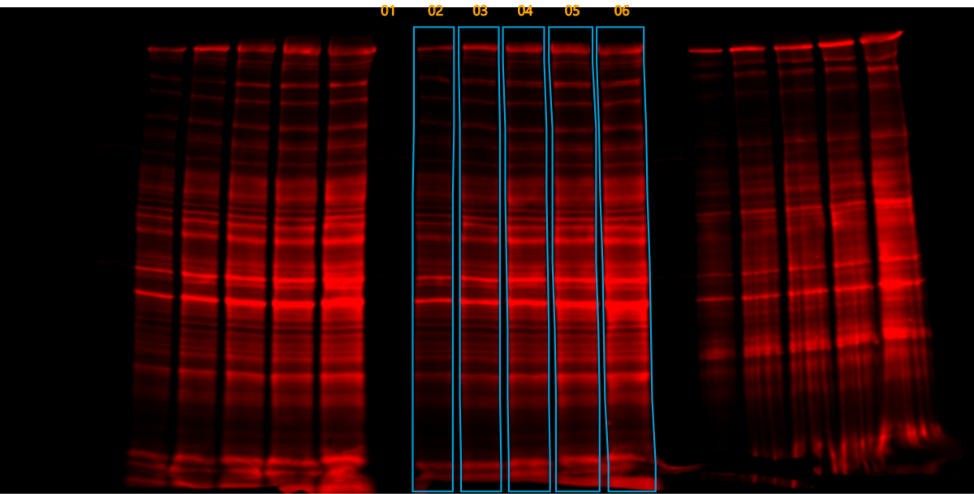 | 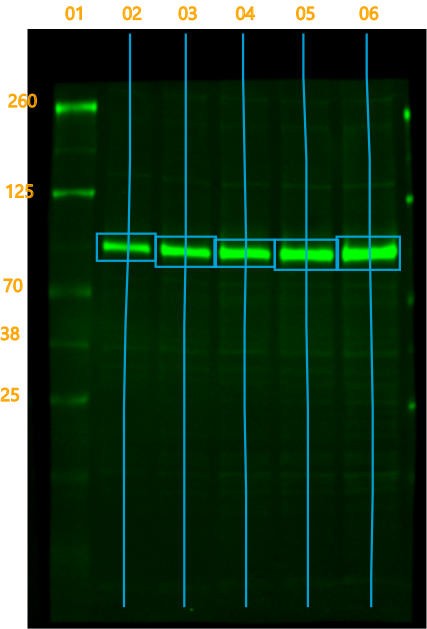 |
| 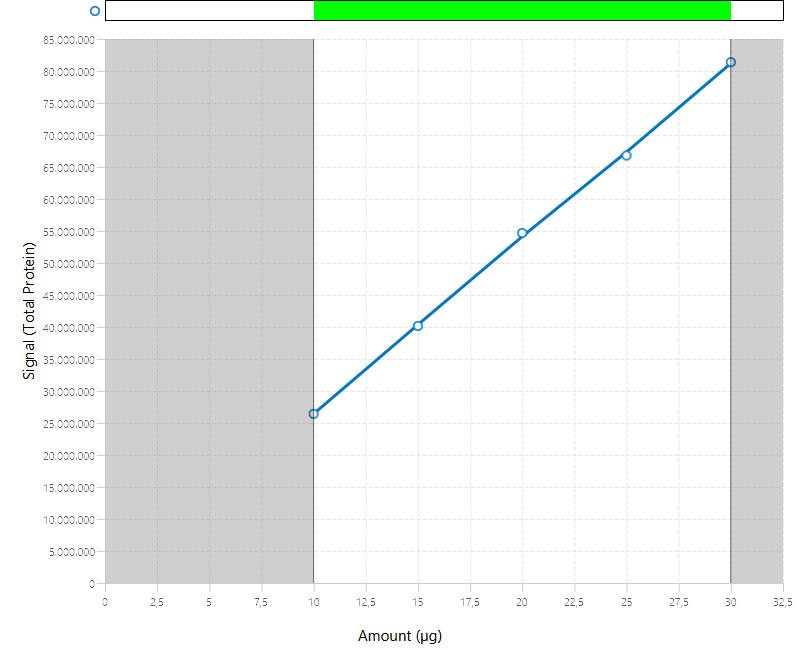 | 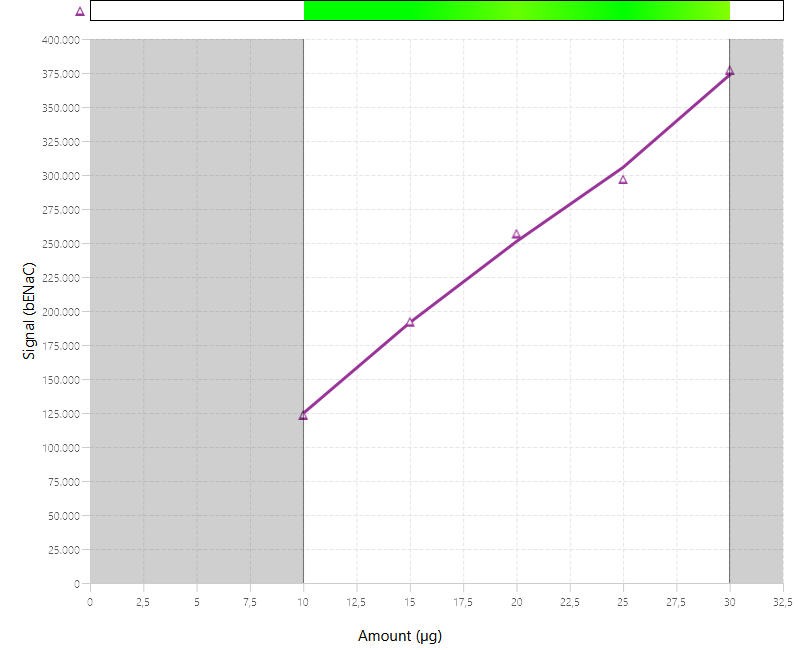 |

**Supplemental Figure 2: Linear range of the used antibodies (continued)**

C Linear range of the bands detected by Anti-γENaC

| Total protein | Anti-γENaC 70 kDa | Anti-γENaC 60 kDa | Anti-γENaC 55 kDa |
| --- | --- | --- | --- |
| 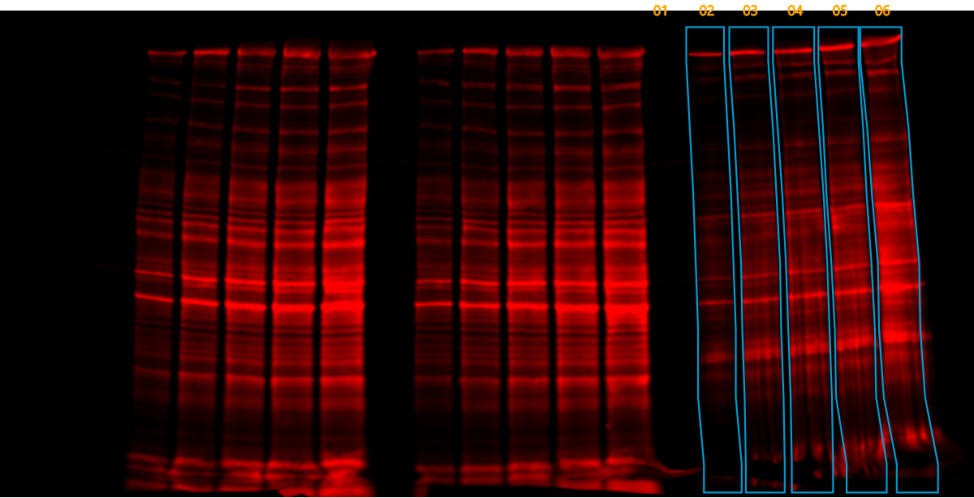 | 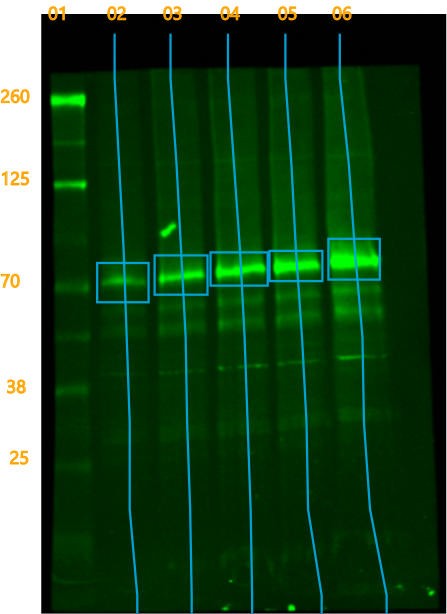 | 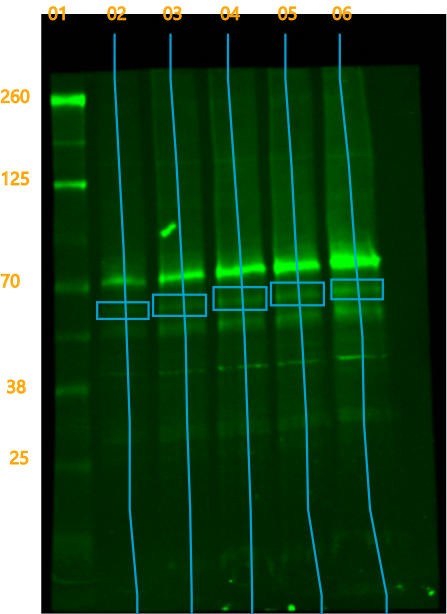 | 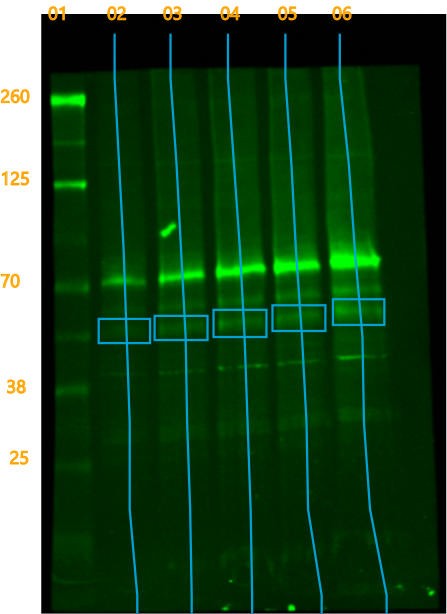 |
| 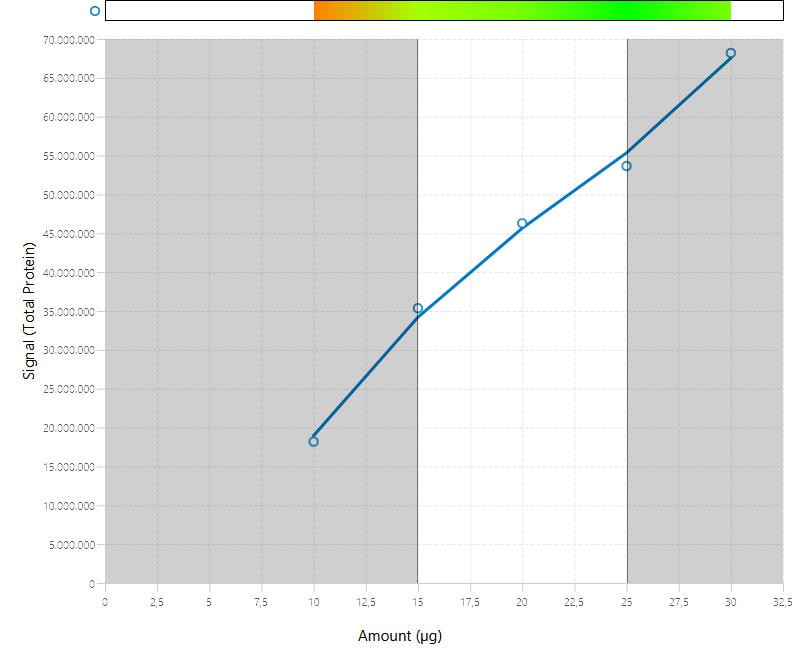 | 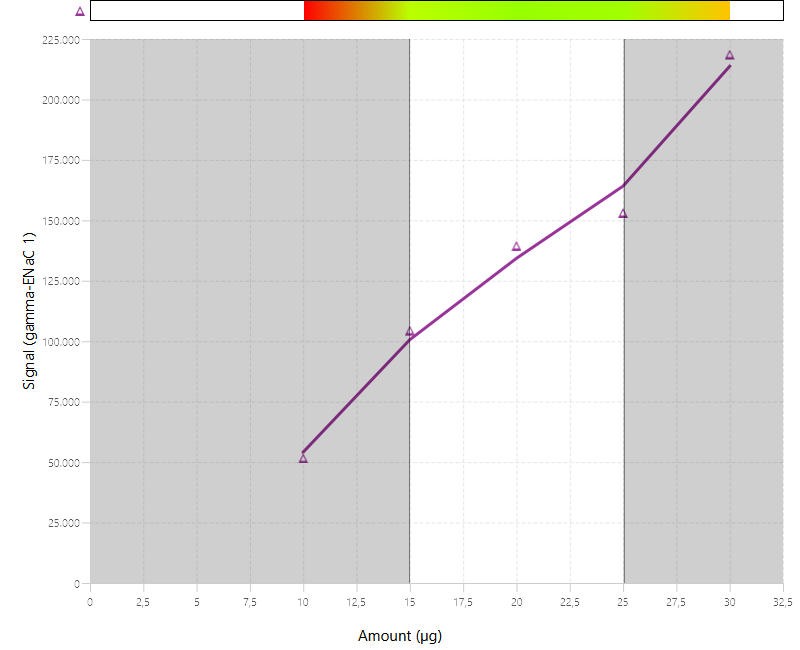 | 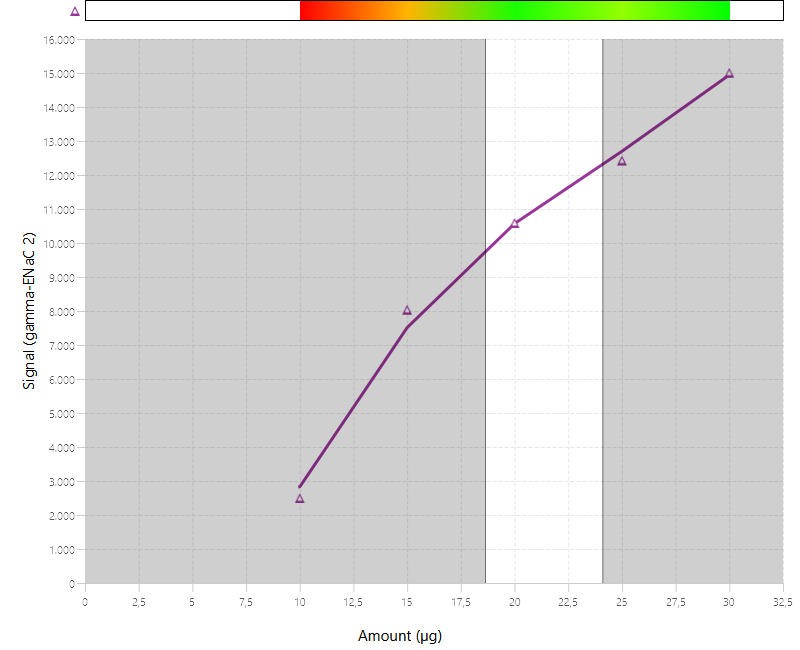 | 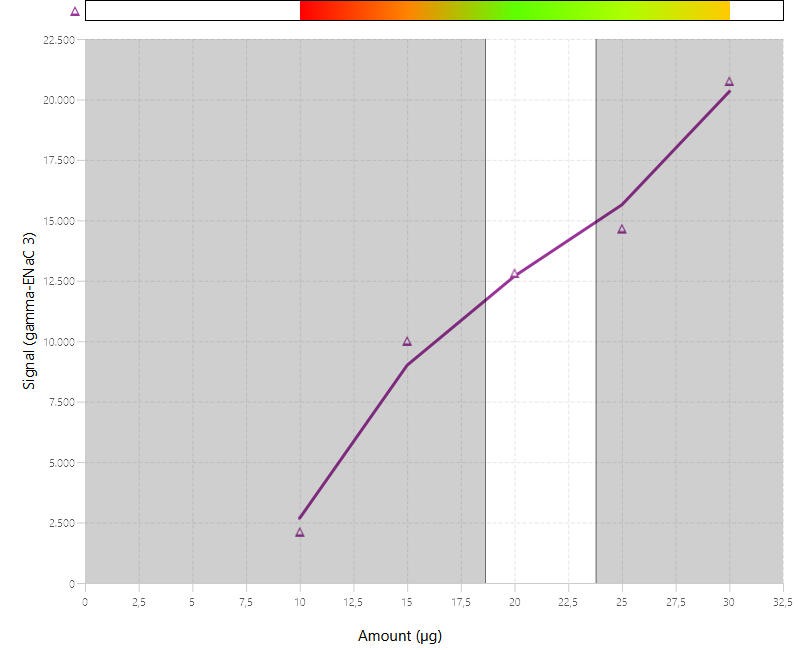 |

**Supplemental Figure 3: Food and fluid intake as well as urine output under a low sodium diet as well as under a 4-day treatment with amiloride in γENaC^wt/wt^ and γENaC^ki/ki^ mice.**

A, B, C Course of food and fluid intake as well as urine output over 24h under a low sodium diet

D, E, F Course of food and fluid intake as well as urine output over 24h under a 4-day treatment with amiloride

^#^ significant difference (p<0.05) between control and intervention in mice of the same genotype (ANOVA with Dunnetts multiple comparison test or Kruskal-Wallis with Dunn’s multiple comparison test) ^*^ significant difference (p<0.05) between genotypes (two-way ANOVA and/or unpaired t-test or Wilcoxon test)) Note that the control values (d,e,g,h) at day 0 were pooled from all experimental series (indicated by larger symbols).


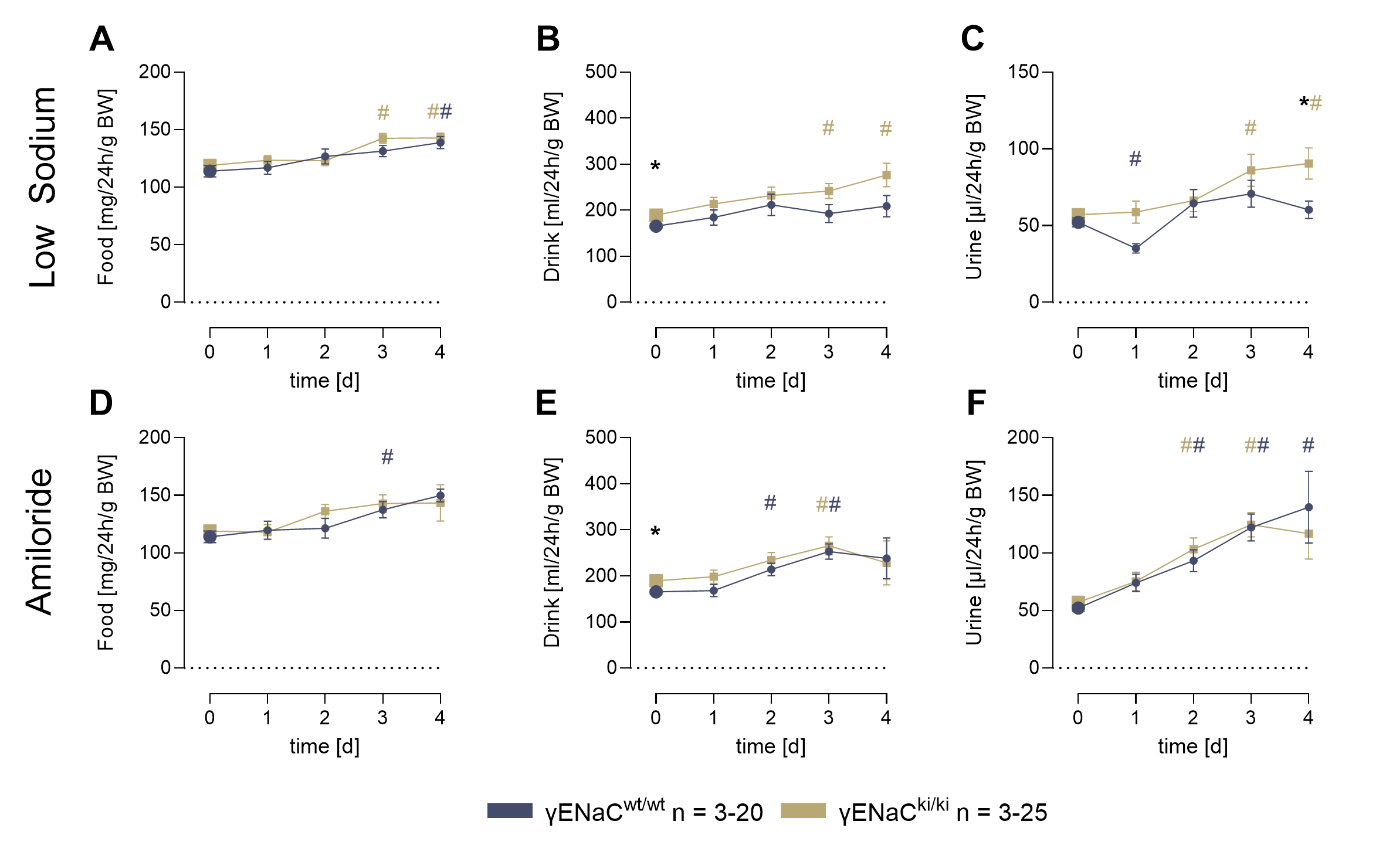


**Supplemental Figure 4: Food (A) and fluid intake (B), calculated sodium intake (C), urinary potassium excretion (D), calculated potassium intake (E) and plasma concentrations of corticosterone (F) before and after induction of experimental nephrotic syndrome in γENaC^wt/wt^ and γENaC^ki/ki^ mice.**

^#^ significant difference (p<0.05) between control and intervention in mice of the same genotype (ANOVA with Dunnetts multiple comparison test or Kruskal-Wallis with Dunn’s multiple comparison test) ^*^ significant difference (p<0.05) between genotypes (two-way ANOVA and/or unpaired t-test or Wilcoxon test)


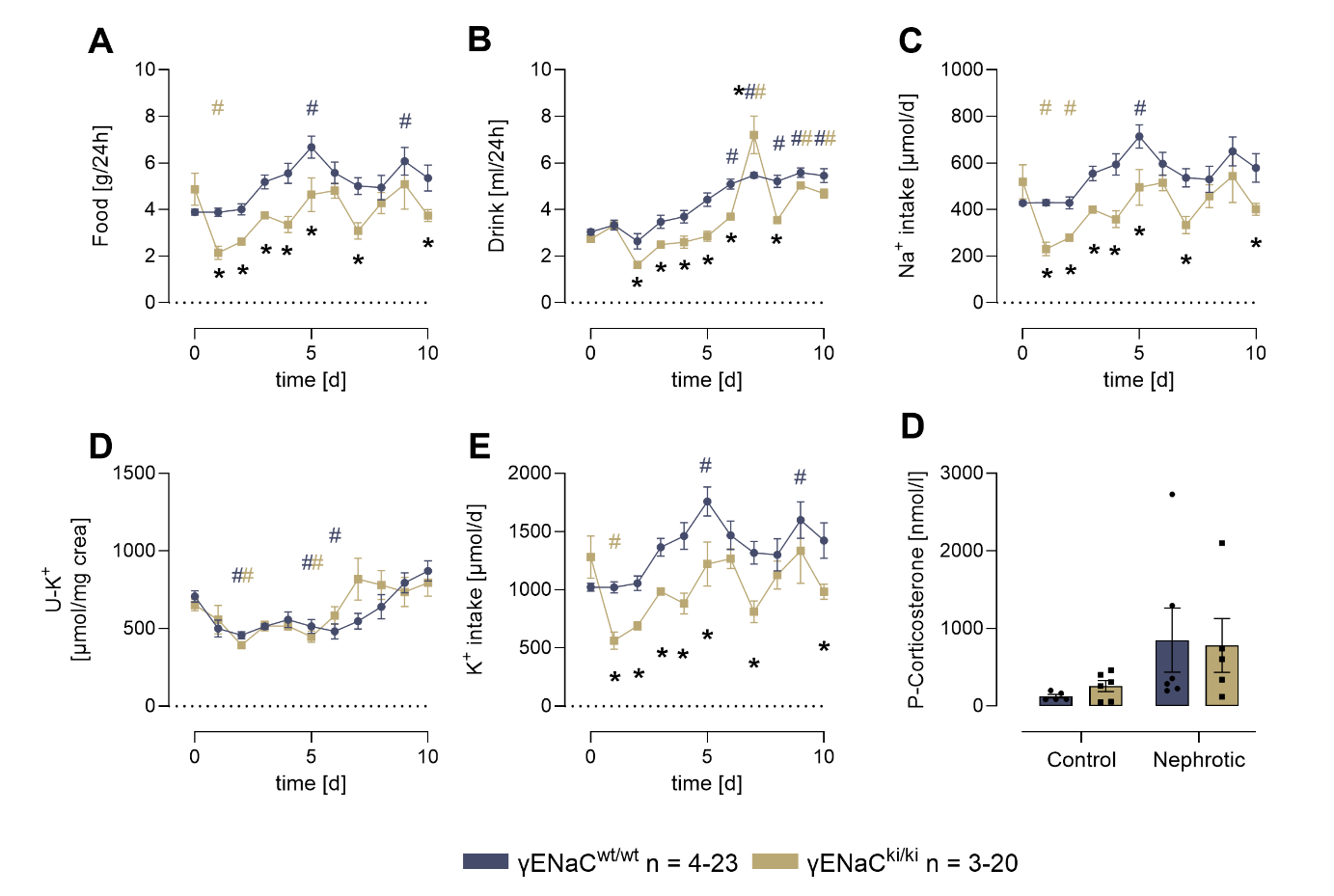

Supplement: Supplementary file 1 — Table S1: Used primers. Table S2: Number of mice used for the experiments and sex distribution. Figure S1: PCR results from genotyping of γENaCwt/wt and γENaCki/ki mice. Figure S2: Linear range of the used antibodies. Figure S3: Food and fluid intake as well as urine output under a low sodium diet as well as under a 4‐day treatment with amiloride in γENaCwt/wt and γENaCki/ki mice. Figure S4: Food and fluid intake, calculated sodium intake, urinary potassium excretion, calculated potassium intake and plasma concentrations of corticosterone before and after induction of experimental nephrotic syndrome in γENaCwt/wt and γENaCki/ki mice. [file APHA-242-e70228-s001.docx]
